# Oral malignant melanoma: A report of two cases with BRAF molecular analysis

**DOI:** 10.3892/ol.2014.2314

**Published:** 2014-07-04

**Authors:** PIER FRANCESCO SOMA, ANGELA PETTINATO, ANNA MARIA AGNONE, CLAUDIO DONIA, GIUSEPPINA IMPROTA, FILIPPO FRAGGETTA

**Affiliations:** 1 Healthcare Burns Unit, Cannizzaro Hospital, Catania 95126, Italy; 2 Pathology Unit, Cannizzaro Hospital, Catania 95126, Italy; 3 Dental Institute, Cannizzaro Hospital, Catania 95126, Italy; 4 Unit of Plastic Surgery, Vittorio Emanuele II Hospital, Castelvetrano (TP) 91022, Italy; 5 Laboratory of Clinical Research and Advanced Diagnostics, IRCCS-CROB, Rionero in Vulture (PZ) 85028, Italy

**Keywords:** oral malignant melanoma, BRAF mutations, therapeutic approaches

## Abstract

Primary oral malignant melanoma is a rare condition, accounting for 1.3–1.4% of all melanomas, usually presenting with an aggressive clinical behavior. The present study reports the clinicopathological findings of two cases of oral malignant melanoma and discusses the epidemiology, diagnosis and current therapeutic approaches for this uncommon condition. In the first case the patient presented with a pigmented lesion located on the lower mucosal lip. The patient showed no nodal metastases and therefore, underwent a wedge resection. After seven months, the patient presented with neck lymph nodes and multiple visceral metastases. Molecular analysis of BRAF, using a pyrosequencing approach, revealed the presence of BRAF V600E mutation. The patient developed multiple visceral metastases, but refused treatment and was lost to follow-up. In the second case, no BRAF V600E mutation was found, but the patient exhibited a pigmented patch in the lower gingival mucosa, which was excised by surgical treatment. The patient was followed up by an oncologist, but did not undergo an additional therapy and is currently alive with no evidence of visceral metastases at one year following the diagnosis.

## Introduction

Melanoma is a malignant tumor that usually involves the skin, however, it may also occur in various extracutaneous sites, including the mucosa (1). Mucosal melanomas, which account for 1.3–1.4% of all melanomas, may arise in the respiratory, gastrointestinal and urogenital tracts with the following incidence levels: Anorectal tract (26.2%), nasal cavity (17.7%), oral cavity (6.5%), vagina (7.4%), penis (3.3%) and urethra (1.8%). Notably, these tumors are clearly distinct from their cutaneous counterparts in their biological behavior, clinical course and prognosis, with no clear risk factors identified at present.

Oral malignant melanoma (OMM) accounts for 0.26–0.5% of all oral malignancies (2), is more commonly diagnosed in older individuals compared with skin melanoma and is extremely rare prior to the age of 20 (median age at diagnosis, 56 years) (3).

Due to its rarity, evidence with regard to treatment recommendations is rare and clinical practice guidelines are largely based on data obtained from case studies and retrospective analyses. The prognosis of OMM is extremely poor, with a reported five-year overall survival rate of 8% (4). Surgery is the preferred treatment for locoregional disease control, and recent diagnostic and therapeutic advancements, including the introduction of immune stimulating antibodies and signal transduction inhibitors, may improve the outcome of metastatic OMM.

In the current study two cases of OMM are reported and the clinicopathological features are presented along with the molecular BRAF analysis. Furthermore, the diagnostic difficulties and treatment options are discussed for this uncommon tumor. Patients provided written informed consent.

## Case reports

### Case one

A 63-year-old male was referred to the Cannizzaro Hospital (Catania, Italy) presenting with a pigmented lesion located on the lower mucosal lip. The patient had been aware of a dark patch for 18 months and received cryotherapy followed by local medical therapy, which was unsuccessful. Surgical excision of the lesion was performed and a final diagnosis of a malignant ulcerated mucosal melanoma, with a diameter of 3.3 mm, which closely extended to the surgical margin, was determined. A clinical re-evaluation revealed no significant cervical lymphadenopathy, and imaging, including chest X-rays and whole body computed tomography scans, revealed no distant metastatic lesions. The patient underwent a wedge resection involving the lower lip and buccal mucosa for the enlargement of the margins, minor salivary gland removal and sentinel submandibular node biopsy, which did not reveal any nodal metastases. Reconstruction was performed using a two-step flap procedure. Interferon-α therapy was then administered.

**Figure 1:**
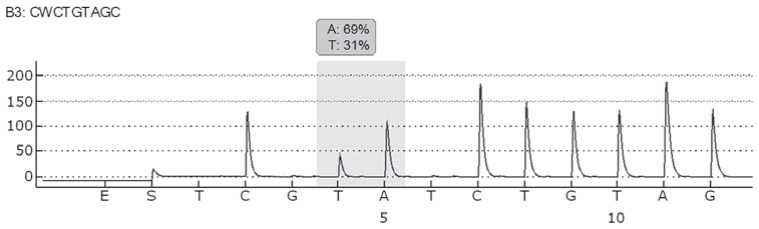
Pyrogram trace obtained following analysis of the sample with a GTG>GAC (p.V600E) mutation in base 2 of codon 600 (nucleotide 1799).

The patient presented seven months later with a hard swelling of the neck lymph nodes. Another surgical intervention was performed as a complete lymph node dissection (level I-II-III-IV), which revealed melanoma involvement in 4/20 nodes. Molecular analysis of BRAF exon 15 codon 600 was performed by pyrosequencing analysis using the Pyromark 24 (Qiagen, Hilden, Germany), according to the manufacturer’s instructions. Molecular analysis revealed the presence of the BRAF V600E mutation in the oral lymph-node metastatic tissue (Fig. 1). The patient then developed multiple visceral metastases, refused treatment and was lost to follow-up.

### Case two

**Figure 2:**
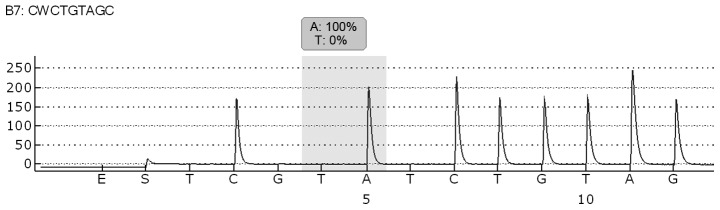
Pyrogram trace obtained following pyrosequencing analysis of the sample. No BRAF mutation was identified at codon 600, exon 15.

A 79-year-old male was found to exhibit a pigmented patch in the lower gingival mucosa during a dental check-up. This lesion increased in size and therefore, the patient was referred to the Cannizzaro Hospital for surgical excision. Upon examination, a black pigmented lesion with irregular borders was observed. The lesion was located on the mouth floor of the lower gingival arch and measured 2.5 cm in diameter. The histological analysis revealed a malignant melanoma, and BRAF molecular analysis of exon 15 codon 600 was performed by pyrosequencing analysis using the Pyromark 24 (Qiagen), according to the manufacturer’s instructions. The molecular analysis revealed no evidence of a BRAF V600E mutation (Fig. 2). The patient was followed-up by an oncologist and no additional therapy was performed. The patient is currently alive with no evidence of disease one year after the diagnosis.

## Discussion

OMM is considered to be an extremely aggressive malignancy due to its tendency to metastasize early during the course of the disease. OMM metastasizes primarily to the lymph nodes, lungs, liver, brain and bones (5). However, in contrast to cutaneous melanoma, which is etiologically associated with sun exposure, the pathogenesis of oral mucosal melanoma remains unclear, although numerous factors have been suggested to exhibit a critical role (6). Ethnicity, as well as cultural and geographical factors may also predispose individuals to the disease, indicated by the fact that Japanese, African, American and Hispanic populations are more commonly affected (1).

Due to the lack of symptoms, particularly in the initial stages of the disease, the diagnosis is often delayed, leading to a poor prognosis and an overall five-year survival rate of 8% (4). Oral melanomas may exhibit different clinical features. The majority occur as pigmented lesions varying from dark brown to blue-black, however, certain oral melanomas may be amelanotic (7). Only a thorough oral examination by a dentist or the patient may lead to the identification of a lesion. The poor prognosis of oral melanomas requires that pigmented lesions of undetermined origin are routinely biopsied.

If possible, surgery with tumor-free margins is the treatment of choice for locoregional disease control. Common sites of occurrence of OMM are the hard palate and maxillary gingiva, however, other oral sites may also be affected, including the mandible, tongue and upper and lower buccal mucosa (7). However, it has become clear that surgical excision of OMM may destroy anatomical structures. Furthermore, radiotherapy has been shown not to improve overall survival, but may reduce the rate of local recurrence. Although melanoma is not highly radiosensitive, patients have occasionally exhibited a good response to radiation therapy, particularly in early melanomas or in melanomas *in situ* (8).

Treatment modalities for advanced disease are similar to those used for cutaneous melanoma. Immunotherapy has been used with limited success, and chemotherapy exhibits a low response rate (9–22). In addition, dacarbazine and IFN-α2b have been used in different combinations, including with bacillus Calmette-Guerin and recombinant interleukin-2, however, results have been disappointing (23). In addition, the BRAF inhibitor, vemurafenib, has not been considered as a common treatment option for patients with mucosal melanoma, as BRAF mutations have been identified much less frequently in patients with mucosal melanoma compared with those arising from cutaneous surfaces (24,25).

However, recent molecular advances have led to the identification of c-KIT as a promising target in OMM, as c-KIT gene alterations have been associated with a frequency of 10–40% in patients with mucosal melanoma, with clinical trials demonstrating the activity of c-KIT inhibitors in the subgroup harboring KIT mutations (26–29).

While mucosal and acral melanomas account for ~65% of all melanomas in Chinese and other Asian populations, in Caucasian populations the predominant location is the trunk and legs, with detection of KIT mutations identified in ≤11% of all melanomas in China (30,31). By contrast, a high prevalence of BRAF mutations (36%) and a lack of KIT mutations were previously found in a study of 11 patients with sinonasal melanoma in Italy (32).

The mitogen-activated protein kinase (MAPK) pathway (RAS/MEK/ERK) is a critical growth cascade in oral mucosal melanoma (33) and it is the most common pathway described in oncogenic events during the progression of melanoma (34,35). One of the molecules that participates in this signal transduction pathway is BRAF, a serine/threonine protein kinase activated by the Ras-GTP protein (36), which incorporates the enzymes RAS (rat sarcoma), RAF, MEK and ERK. The MAPK pathway is downstream of the receptor tyrosine kinases, cytokines and G protein-coupled receptors, leading to cell growth, survival and differentiation. A novel therapeutic approach has been suggested for advanced-stage cutaneous melanoma, whereby a BRAF mutation at codon 600 has been identified, leading to a novel approach for drug development in the advanced setting (35).

V600E, a protein substitution of valine for glutamic acid at position 600 (Val600Glu), is the most common BRAF mutation observed in cutaneous melanoma, which consists of a T1799A transversion mutation in exon 15 of this gene. This mutation accounts for >90% of all BRAF mutations detected thus far in cutaneous melanoma (36,37), leading to ERK activation and a subsequent proliferation and survival advantage in melanoma cells. Another molecule that leads to the activation of MAPK is RAS, which is encoded by the RAS gene, consisting of HRAS, KRAS and NRAS. Frequently, NRAS and BRAF mutations have been observed in cutaneous melanoma and in subsets of mucosal melanoma (38–40). In addition, the MAPK pathway may be triggered by the activation of c-KIT, leading to the induction of signaling proteins, essentially stuck in the ‘on’ position, resulting in uncontrolled cell proliferation and survival (41). Mutations in the c-KIT gene, along with BRAF mutations, in part, considered to be involved in the mechanism of development and progression of melanoma, have been identified in mucosal melanoma, which not only implicates BRAF, but also c-KIT, as a promising molecular target (42–44). Thus, drug therapies have been developed to inhibit these mutations, preventing tumor proliferation. One targeted therapy is vemurafenib, which was approved by the US Food and Drug Administration in August 2011 for the treatment of patients with unresectable or metastatic melanoma with BRAF V600E (45). Vemurafenib is a selective inhibitor of the activated form of the BRAF serine-threonine kinase enzyme, with a low molecular weight and oral availability.

However, melanoma is widely known to be a molecularly heterogeneous disease, exhibiting variation at the genetic level. Furthermore, a molecular classification system identifies four distinct genetic types of melanoma, including melanoma arising from non-chronically sun-damaged skin, melanoma arising from chronically sun-damaged skin, melanoma arising from acral surfaces and melanoma arising from mucosal surfaces. These types are all characterized by unique combinations of genome-wide aberrations in DNA copy number and oncogenic alterations.

The current study presents a case of OMM harboring the BRAF V600E mutation, and highlights the importance of testing patients with oral melanoma for the presence of BRAF mutations.

In conclusion, the present study reports the clinicopathological findings of two notable cases of oral malignant melanoma and discusses the epidemiology, diagnosis and current therapeutic approaches. Molecular analysis of BRAF revealed the presence of BRAF V600E mutation only in the first case of a patient with a more aggressive disease than the second case with no BRAF V600E mutation. Despite BRAF mutations appearing to be frequently involved in the pathogenesis and progression of cutaneous malignant melanoma, recently, several studies have demonstrated a low incidence of BRAF mutations in melanoma arising from non-hair-bearing skin that is relatively protected from ultraviolet light damage, in melanoma arising from mucosa that is completely sun protected and in oral malignant melanoma (46). Therefore, BRAF mutations must not be disregarded in oral malignant melanoma, underlining the importance of the molecular analysis of BRAF mutations for patients affected by this rare disease subtype.

## References

[1] Moreira RN, Santos CR, Lima NL, Verli FD, Marinho SA (2010). Oral and cutaneous melanoma: similarities and differences. J Clin Med Res.

[2] Yang X, Ren GX, Zhang CP (2010). Neck dissection and post-operative chemotherapy with dimethyl triazeno imidazole carboxamide and cisplatin protocol are useful for oral mucosal melanoma. BMC Cancer.

[3] Barker BF, Carpenter WM, Daniels TE (1997). Oral mucosal melanomas: the WESTOP Banff workshop proceedings. Western Society of Teachers of Oral Pathology Oral Surg Oral Med Oral Pathol Oral Radiol Endod.

[4] Bachar G, Loh KS, O’Sullivan B (2008). Mucosal melanomas of the head and neck: experience of the Princess Margaret Hospital. Head Neck.

[5] Guevara-Canales JO, Gutiérrez-Morales MM, Sacsaquispe-Contreras SJ, Sánchez-Lihón J, Morales-Vadillo R (2012). Malignant melanoma of the oral cavity. Review of the literature and experience in a Peruvian Population. Med Oral Patol Cir Bucal.

[6] Silverman S (2003). Etiology and predisponing factors. Oral Cancer.

[7] Hashemi Pour MS (2008). Malignant melanoma of the oral cavity: a review of literature. Indian J Dent Res.

[8] Jahanshahi P, Nasr N, Unger K, Batouli A, Gagnon GJ (2012). Malignant melanoma and radiotherapy: past myths, excellent local control in 146 studied lesions at Georgetown University, and improving future management. Front Oncol.

[9] Greenberg MS, Glic KM (2003). Pigmented lesions of the oral mucosa. Burket’s Oral Medicine.

[10] Prabhu SR, Wilson DF, Daftary DK (1992). Oral and salivary gland neoplasms in tropical populations. Oral Diseases in the Tropics.

[11] Neville BW, Damm D, Allen CM, Bouquot JE (2002). Epithelial pathology. Oral and Maxillofacial Pathology.

[12] van der Waal RI, Snow GB, Karim AB, van der Waal I (1994). Primary malignant melanoma of the oral cavity: a review of eight cases. Br Dent J.

[13] Robertson GR, DeFiebre BK, Firtell DN (1979). Primary malignant melanoma of the mouth. J Oral Surg.

[14] Rapidis AD, Apostolidis C, Vilos G, Valsamis S (2003). Primary malignant melanoma of the oral mucosa. J Oral Maxillofac Surg.

[15] Cebrián Carretero JL, Chamorro Pons M, Montesdeoca N (2001). Melanoma of the oral cavity. Review of the literature. Med Oral.

[16] Lopez-Graniel CM, Ochoa-Carrillo FJ, Meneses-García A (1999). Malignant melanoma of the oral cavity: diagnosis and treatment experience in a Mexican population. Oral Oncol.

[17] Rapini RP (1997). Oral melanoma: diagnosis and treatment. Semin Cutan Med Surg.

[18] Wood NK, Goaz PW (1997). Solitary red lesions; intraoral brownish, bluish, or black conditions. Differential Diagnosis of Oral and Maxillofacial lesions.

[19] Liebross RH, Morrison WH, Garden AS, Ang KK (1997). 50 Mucosal melanoma of the head and neck. Int J Radiol Oncol.

[20] Doval DC, Rao CR, Saitha KS (1996). Malignant melanoma of the oral cavity: report of 14 cases from a regional cancer centre. Eur J Surg Oncol.

[21] Nandapalan V, Roland NJ, Helliwell TR (1998). Mucosal melanoma of the head and neck. Clin Otolaryngol Allied Sci.

[22] Ord RA, Blanchaert RH (1999). The dentist’s role in diagnosis, management, rehabilitation and prevention. Oral Cancer.

[23] Takagi M, Ishikawa G, Mori W (1974). Primary malignant melanoma of the oral cavity in Japan. With special reference to mucosal melanosis. Cancer.

[24] Curtin JA, Fridlyand J, Kageshita T (2005). Distinct sets of genetic alterations in melanoma. N Engl J Med.

[25] Maldonado JL, Fridlyand J, Patel H (2003). Determinants of BRAF mutations in primary melanomas. J Natl Cancer Inst.

[26] Curtin JA, Busam K, Pinkel D, Bastian BC (2006). Somatic activation of KIT in distinct subtypes of melanoma. J Clin Oncol.

[27] Beadling C, Jacobson-Dunlop E, Hodi FS (2008). KIT gene mutations and copy number in melanoma subtypes. Clin Cancer Res.

[28] Kong Y, Si L, Zhu Y (2011). Large-scale analysis of KIT aberrations in Chinese patients with melanoma. Clin Cancer Res.

[29] Hodi FS, Corless CL, Giobbie-Hurder A (2013). Imatinib for melanomas harboring mutationally activated or amplified KIT arising on mucosal, acral, and chronically sun-damaged skin. J Clin Oncol.

[30] Chi Z, Li S, Sheng X (2011). Clinical presentation, histology, and prognoses of malignant melanoma in ethnic Chinese: a study of 522 consecutive cases. BMC Cancer.

[31] Shoo BA, Kashani-Sabet M (2009). Melanoma arising in African-, Asian-, Latino- and Native-American populations. Semin Cutan Med Surg.

[32] Turri-Zanoni M, Medicina D, Lombardi D (2013). Sinonasal mucosal melanoma: Molecular profile and therapeutic implications from a series of 32 cases. Head Neck.

[33] Govindarajan B, Bai X, Cohen C (2003). Malignant transformation of melanocytes to melanoma by constitutive activation of mitogen-activated protein kinase kinase (MAPKK) signaling. J Biol Chem.

[34] Satyamoorthy K, Li G, Gerrero MR (2003). Constitutive mitogen-activated protein kinase activation in melanoma is mediated by both BRAF mutations and autocrine growth factor stimulation. Cancer Res.

[35] Solit DB, Garraway LA, Pratilas CA (2006). BRAF mutation predicts sensitivity to MEK inhibition. Nature.

[36] Davies H, Bignell GR, Cox C (2002). Mutations of the BRAF gene in human cancer. Nature.

[37] Platz A, Egyhazi S, Ringborg U, Hansson J (2008). Human cutaneous melanoma; a review of NRAS and BRAF mutation frequencies in relation to histogenetic subclass and body site. Mol Oncol.

[38] Wong CW, Fan YS, Chan TL, Cancer Genome Project (2005). BRAF and NRAS mutations are uncommon in melanomas arising in diverse internal organs. J Clin Pathol.

[39] Poynter JN, Elder JT, Fullen DR (2006). BRAF and NRAS mutations in melanoma and melanocytic nevi. Melanoma Res.

[40] Saldanha G, Potter L, Daforno P, Pringle JH (2006). Cutaneous melanoma subtypes show different BRAF and NRAS mutation frequencies. Clin Cancer Res.

[41] Lennartsson J, Jelacic T, Linnekin D, Shivakrupa R (2005). Normal and oncogenic forms of the receptor tyrosine kinase kit. Stem Cells.

[42] Rivera RS, Nagatsuka H, Gunduz M (2008). C-kit protein expression correlated with activating mutations in KIT gene in oral mucosal melanoma. Virchows Arch.

[43] Curtin JA, Busam K, Pinkel D, Bastian BC (2006). Somatic activation of KIT in distinct subtypes of melanoma. J Clin Oncol.

[44] Beadling C, Jacobson-Dunlop E, Hodi FS (2008). KIT gene mutations and copy number in melanoma subtypes. Clin Cancer Res.

[45] Smyth E, Carvajal R (2011). Treatment of Metastatic Melanoma: A New World Opens. Skin Cancer Foundation Journal.

[46] Buery RR, Siar CH, Katase N (2011). NRAS and BRAF mutation frequency in primary oral mucosal melanoma. Oncol Rep.

